# FORMAL AND DAILY CARE DECISION-MAKING INVOLVEMENT IN AFRICAN-AMERICAN DEMENTIA DYADS

**DOI:** 10.1093/geroni/igz038.3335

**Published:** 2019-11-08

**Authors:** Kalisha Bonds, MinKyoung Song, Karen Lyons, Martha Driessnack

**Affiliations:** 1 Emory University, Atlanta, Georgia, United States; 2 Oregon Health & Science University, Portland, Oregon, United States; 3 Boston College William F. Connell School of Nursing, Chestnut Hill, Massachusetts, United States

## Abstract

Decision-making involvement (e.g., verbal and/or nonverbal communication) of persons with dementia (PWD) has been associated with quality of life of PWDs and their caregivers, underscores personhood, and reduces ethical dilemmas for caregivers regarding the PWD’s care. Yet, no study has explored the decision-making involvement in formal and daily care of both members of African-American dementia dyads (i.e., African-American PWDs and their African-American caregivers), limiting our understanding of how these dyads navigate decision-making during the dementia trajectory. This study took a closer look through in-depth, semi-structured interviews with African-American dementia dyads as they reflected on their decision-making surrounding formal and daily care. A pilot study of five dyadic interviews, each averaging 45 minutes, was completed. We used a combination of quantitative content analysis, decision-making matrices and I-poems created from I-statements of the dyad regarding their decision-making involvement. Decision-making matrices (i.e., diagrams of the degree of sharing, the balance of power within the dyad, and the final decision maker in formal and daily care) were constructed across interviews. The pairing of traditional analyses with the novel use of I-poems traces participants’ sense of self, ensuring their voice is retained. There was agreement within all five dyads regarding the final decision maker(s) in formal and daily care. Between dyads, daily decision-making involvement was led by African American PWDs; whereas, formal care decision-making involvement of African American PWDs varied. Findings highlight the importance of a deeper understanding of formal and daily care decision-making involvement within and between African-American dementia dyads and potential clinical implications.

